# Sick and Tired—Sociodemographic and Psychosocial Characteristics of Asylum Seekers Awaiting an Appointment for Psychotherapy

**DOI:** 10.3390/ijerph182211850

**Published:** 2021-11-12

**Authors:** Ulrich Trohl, Karoline Wagner, Vivian Kalfa, Sarah Negash, Andreas Wienke, Amand Führer

**Affiliations:** 1 Institute for Medical Epidemiology, Biometrics and Informatics, Interdisciplinary Center for Health Sciences, Medical School of the Martin-Luther-University Halle-Wittenberg, 06112 Halle (Saale), Germany; karoline.wagner@uk-halle.de (K.W.); sarah.negash@uk-halle.de (S.N.); andreas.wienke@uk-halle.de (A.W.); amand-gabriel.fuehrer@uk-halle.de (A.F.); 2 Psychosocial Centre for Refugees and Victims of Torture, 06108 Halle (Saale), Germany; kalfa@psz-sachsen-anhalt.de

**Keywords:** asylum seekers, mental health, secondary data analysis, health care utilization

## Abstract

Background: An EU directive holds the EU member states responsible for implementing the provision of health care for asylum seekers. However, current literature indicates insufficient care for asylum seekers in the German health system. This article aims to characterize the situation of the client population on the waiting list of a psychosocial center (PSZ). Methods: We conducted a retrospective observational study based on client files in Halle (Saale), Germany. We included 437 adults who were on the PSZ waiting list between 2016 and 2019. Questionnaires that collected information on the clientele at two different times were analyzed. Results: The average waiting time for psychotherapy was 50 weeks. In total, 85.6% of the 188 respondents reported sleep disorders (*n* = 161), 65.4% of clients reported pain (*n* = 123) and 54.8% suicide attempts/suicidal thoughts (*n* = 54). In the 16-week waiting period in which the clients waited for an initial appointment with a psychologist, the residence status deteriorated in 21.3% (*n* = 40). Conclusion: Improving asylum seekers’ access to the German health system is urgently needed in order to prevent unnecessary suffering in the future and to comply with EU law.

## 1. Introduction

By the end of 2019, 79.5 million people had been forcibly displaced worldwide. They are fleeing war, persecution and human rights violations [1]. Accordingly, there has also been a considerable increase in immigration to Germany in recent years. Given the etiology of mental disorders, it is not surprising that the prevalence of mental illness among refugees is high [2,3]. In the literature on asylum seekers in Germany, a prevalence between 28–75% for depression, 45% for anxiety disorders and up to 41% for PTSD are described for different cohorts of asylum seekers [4,5]. A study with samples from facilities that accommodate asylum seekers at the beginning of their stay in Germany found an even higher prevalence [6].

Therefore, among refugees there are people whose mental illness creates severe suffering and makes psychotherapy urgently necessary. The therapeutic needs of these people differ from those of the general population and challenge the German health system to deal with language barriers and transcultural issues [7]. Accordingly, the health reports of the German Association of Psychosocial Centres for Refugees and Victims of Torture (*BafF*) have been warning each year since 2014 that asylum seekers’ care is inadequate [8].

In order to ensure adequate medical care for asylum seekers in their host countries, this issue was addressed in 2013 by Directive 2013/33/EU of the European Parliament and of the Council [9], which states in Article 19:

“Member States shall provide necessary medical or other assistance to applicants who have special reception needs, including appropriate mental health care where needed”.

In addition, it specifies that “Member States shall take into account the specific situation of vulnerable persons such as […] persons with mental disorders and persons who have been subjected to torture, rape or other serious forms of psychological, physical or sexual violence, such as victims of female genital mutilation, in the national law implementing this Directive”. (Article 21).

In this context, the term “appropriate mental health care” does not only refer to an ethical ambition by the member states of a community of values, or to the professional ethics of caretakers and individual citizens: Beyond that, health is also a human right and as such “the basis for freedom, justice and peace, […] the prevention of discrimination, oppression, avoidable suffering and harm” [10]. It also goes beyond financial aspects that make quick and effective first aid desirable for payers and authorities [11]. The EU raises this issue to a higher legal level and with the Directive 2013 created a legal basis that is binding for the member states.

### 1.1. Asylum Seekers‘ Access to Health Care in Germany

Asylum seekers are at a substantially increased risk of mental health problems. Still, their access to medical care is subject to legal restrictions in Germany [11,12].

In the federal state of Saxony-Anhalt in particular, asylum seekers do not automatically receive an electronic health insurance card that would put them on an (almost) equal footing with patients with statutory health insurance covering around 88% of the German population. Instead, they must contact the local social services office in the event of illness. Upon application, this office can issue a treatment voucher that is only valid for a specific time period and for previously determined services [13]. The asylum seeker has to manage this process at a time of illness, which often complicates and lengthens the treatment process in addition to known barriers such as language difficulties, legal uncertainties and lack of mobility. The treating physicians, on the other hand, have to enter into a reimbursement process that differs from standard care and is less familiar to them [14,15]. Studies suggest that this intentional restriction of access to the health system is ultimately even more costly [16]. Superfluous bureaucracy has to be managed and the threat of chronification complicates treatments. Early, low-threshold and outpatient treatments of the acute illness are cheaper for the health care service than emergencies provoked by this restriction [11].

As a result of this situation, many asylum seekers have no access to timely and guideline-adherent mental health care. Previous research by our working group has shown that there is a substantial supply gap for asylum seekers in the outpatient sector [17]. As a result, they often have to resort to low-threshold emergency departments [18], which cannot provide the continuous long-term care mental disorders require. Further literature also shows that the German healthcare system has not sufficiently opened up to the needs of asylum seekers [19].

Therefore, many asylum seekers who receive no care from regular health care institutions seek help outside the health care system funded by health insurance and are treated by civil society organizations. Most prominent among these are the Psychosocial Centers for Refugees and Torture Victims, which exist in 38 cities in Germany and offer specialized services for transcultural psychotherapy with a focus on traumatized patients. One of these institutions is the Psychosocial Center for Migrants (PSZ) in Halle (Saale).

Hitherto, little is known about the medical histories of those who resort to treatment in the PSZ because they do not have access to treatment in the regular health care system.

### 1.2. Aim of the Study

Since presumably the PSZ is the only option for most asylum seekers with mental complaints, this study aims to further characterize the PSZ’s client population. We hereby aim to describe the demographics of this population, the length of the waiting time and the PSZ’s services that have been used. In addition, the psychopathologies and diagnoses as well as aggravating conditions of the clients on the one hand and their resources on the other hand are descriptively examined.

## 2. Materials and Methods

This study is based on a secondary data analysis of clients’ charts. Since there are very few literatures on this particular study population and generating hypotheses concerning differences between subgroups would have been difficult, we hereby used an exploratory approach to describe the PSZ’s client population.

### 2.1. Setting

The ‘Psychosocial Center for Migrants in Saxony-Anhalt’ is an institution run and financed by a non-profit company and is dedicated to provide psychosocial support and psychotherapy for refugees. It is an outpatient center and offers psychological counselling, psychotherapy, expert opinions and social counselling. These services are free of charge for the clients and are offered irrespective of their residence status. Psychotherapy is carried out with a focus on refugees who have survived persecution, violence and torture. In addition, cultural and linguistic backgrounds are given special consideration in this setting [8].

### 2.2. Data Source and Collection

This analysis builds on secondary data from the PSZ Halle. It uses information from the clients’ files routinely collected at two points in time at the beginning of the registration process: The first source of information is a registration form completed by the applicants themselves and serves to register the client. We analyzed the registration forms of 437 clients. The second source are records from the assessment interview. This is conducted by psychologists (and sometimes social workers) and includes a more detailed assessment of the client’s needs. Here we analyzed the charts of 188 clients who got an appointment for the assessment interview within our observation period.

Data from the period of 1 January 2016 to 31 December 2019 were selected for evaluation since uniform questionnaires were used during this period. All questionnaires were originally available on paper and handwritten.

The data collection was carried out from August to November 2020. The medical records were located directly on site of the PSZ. The data were entered anonymized into a pretested entry form. Most variables were entered already in categorized form, while some variables were entered as text and categorized later on.

### 2.3. Inclusion and Exclusion Criteria

This study included all adult persons who indicated their need for the PSZ’s support during the above-mentioned period by submitting a completed registration form.

### 2.4. Variables

In order to reflect the needs of clients, their current situation and their medical care, variables from the two above-mentioned questionnaires were analyzed.

The registration form provides an insight into the situation at the time of application. We were interested in demographic data and medical history such as acute symptoms, diagnoses, contacts with the health system and information on emergency situations. It also contains information on the residence status, particular concerns of the clients, the professional who referred them to the PSZ and whether they possess an electronic health insurance card.

The assessment interview is used to assess the specific needs. In most cases, this requires an interpreter. In this intercultural setting, these interpreters play a vitally important role. Ilkilic also highlights the ethical importance of interpreters [20]. The interview provides additional data on torture experiences, the professional’s assessment of the urgency of the situation, previous contact to physicians, current medication and social support network. The psychologists also document disorder-relevant psychopathological symptoms and once again collect information on residence status. In order to support clients with urgent concerns during waiting time, they are given contacts for further assistance. These contacts are also documented.

Since the registration form is mostly filled in by medical laypersons and there are several items in both questionnaires where text can be entered, the subsequent interpretation of the results requires a classification. We categorized these data in the following method:

1. When depression as such was reported as a ‘reason for consultation’, the term ‘depression’ was adopted. On the other hand, when symptoms of depression were listed (such as sadness, listlessness, anhedonia), we summarized this information as ‘depressiveness’. Since they were lay data and differentiation was not possible in hindsight, the diagnosis of post-traumatic stress disorder (PTSD) and the less specific term ‘trauma’ were summarized as ‘trauma’. The resulting categories of reasons for searching therapeutic help are later on presented as “complaints” at the time of the application.
2. The documented ‘diagnoses’ were encoded according to the ICD-10. If only the information ‘depression’ was given, this was recorded as F32.9 (=Depressive episode, unspecified). If diagnoses were stated with the German prefix ‘V.a.’, indicating a suspected diagnosis, this information was listed separately.
3. Since the majority of clients could not indicate a specific drug when asked about their prescribed ‘medications’, classification was carried out according to less detailed drug groups.
4. During the assessment interview, there was no explicit question about ‘torture experiences’. However, if the clients reported such experiences unprompted, this was documented. In addition to the answer options yes and no, the psychologist was able to document the information about torture as ‘unclear’.
5. In the course of the assessment interview, the psychologist specifically asked for ‘disorder-relevant psychopathological symptoms’. In addition to less specific items, such as sleep disorders, pain, anxiety, restlessness and appetite disorders, clear psychiatric symptoms such as suicidal- and self-injuring behavior, aggressiveness, drug use and hallucinations were also explored. During the assessment interview, these topics are explored anamnestically only, there is no standardized diagnostic at this point. We therefore report them below as “disorder-relevant psychopathological symptoms” and differentiate them from “diagnoses”, which have been assigned by physicians prior to registration with the PSZ.
6. In order to assess the extent to which the ‘residence status of the clients changed’, we divided them into secure, precarious and at-risk status. When classifying, we considered legal aspects as well as the psychosocial stress associated with different statuses and their biographical implications from the asylum seeker’s point of view. We classified residence permits (in German: *Aufenthaltserlaubnis*) as ‘secure’. ‘Precarious statuses’ were temporary suspension of deportation (*Duldung*) and temporary residence permit for the time of the asylum request (*Aufenthaltsgestattung*). We defined a situation as ‘at-risk’ when a deportation was imminent, a title had expired or was rejected, a person was without residence status in Germany or had to live in church sanctuary. We compare these categories from the time of registration with those for the assessment interview on the level of the individual.
7. From our data, two ‘waiting times’ could be determined. Firstly, the waiting period from the registration to the assessment interview. Secondly, the waiting time from the assessment interview to the beginning of psychotherapy. In order to classify the waiting times, we compared them with the average waiting time for psychotherapy of clients with regular statutory health insurance in the state of Saxony-Anhalt.

### 2.5. Statistical Analysis

Two researchers independently categorized text information. These categories were then compared, and discrepancies discussed until consensus was reached. Then the resulting categories were analyzed alongside the other variables using descriptive statistics.

We report absolute and relative frequencies. For subgroup analyses, stratified analyses according to gender and country of origin were conducted. Since this study followed an exploratory approach, we followed established guidelines [21,22] and did not conduct significance tests for differences between subgroups.

All analyses were performed in SAS (Cary, North 219, CA, USA).

### 2.6. Ethical Considerations

Prior to data collection from the clients’ charts, ethical approval was granted by the Ethics Committee of Martin-Luther-University Halle-Wittenberg (Protocol Code: 2020-068).

## 3. Results

In order to summarize the results, the demographic data and the situation of the clients at the time of registration are presented in the following. We then turn to the clients’ situation during the assessment interview. Hereby, different denominators are used: Variables collected in the registration form are referenced to a total of 437 persons, whereas for variables originating from the assessment interview the denominator is 188 persons. Of those, 81 proceeded to receive psychotherapy within the observed period of time.

On average, there are 16 weeks between the time of registration and the assessment interview (“time until assessment”). Changes that occurred during this period are presented in the last section of the results. After assessment, the average waiting time until the commencement of therapy was 50 weeks.

### 3.1. Demography

This study included 437 registered clients of the PSZ (men: *n* = 266, 60.9%; women: *n* = 171, 39.1%). Clients came from 39 different countries, with Afghanistan (*n* = 139, 31.8%) being the most common country of origin, followed by Syria (*n* = 56, 12.8%), Iran (*n* = 41, 9.4%), Russia (*n* = 22, 5.0%) and Somalia (*n* = 20, 4.6%). For seven people (1.8%) there was no information on the country of origin. The age distribution was roughly the same as the distribution of asylum seekers in Germany by age group in 2020. The median age was 30.7 years (min: 18 years, max: 61 years). The most common language was Persian/Farsi/Dari (*n* = 183, 41.9%) followed by English (*n* = 83, 19.0%), German (*n* = 75, 17.2%) and Arabic (*n* = 71, 16.3%). In 82.4% of cases, an interpreter was deemed to be required for psychotherapy. In total, 223 people (51.0%) lived in Halle. The remaining applicants resided in smaller district towns in the state of Saxony-Anhalt.

The demographic data on age and country of origin in the study population largely correspond to the distribution in the general refugee population in Germany and Europe [23]. However, the group of Syrian asylum seekers is proportionally underrepresented. This could be due to the fact that Syrian refugees receive a positive asylum decision more quickly and are thus entitled to regular health care.

More information on demography is given in Table 1.

### 3.2. Situation at the Time of Application

A total of 437 client files were available to evaluate the situation at the time of the application.

Complaints

As the main complaint, most clients specified insomnia (*n* = 225, 51.5%). Anxiety (*n* = 143, 32.7%), trauma/PTSD (*n* = 84, 19.2%) and headaches (*n* = 81, 18.5%) were other complaints that were frequently stated. Multiple answers could be given in this category.

In total, 47.4% of clients considered their complaints to be an emergency (*n* = 207). As a rationale for this emergency, suicidal ideations or attempted suicide or self-injury (*n* = 81, 39.1%) are cited often. Other common reasons were acute stress situations (*n* = 28, 13.5%), threatening deportation (*n* = 21, 10.1%) and social indications (*n* = 20, 9.7%).

When stratifying the complaints and their treatment’s urgency according to gender and country of origin, no relevant differences between subgroups were found.

More details are given in Table 2 and Table 3.

Diagnoses

Asked about previously diagnosed diseases, clients reported primarily post-traumatic stress disorder (44.7% of those stating a response and 14.4% of the total cohort). Overall, 64.5% (or 20.8%, respectively) of the applicants had a disease in the category ‘reaction to severe stress, and adjustment disorder’. In total, 34.7% (or 11.1%) reported illnesses in the ‘depressive episode’ category.

More details are given in Table S1.

Treatments

A total of 190 clients indicated on their application form that they were already receiving medical treatment (43.5%). Of those, 30.6% (*n* = 58), reported contacts with outpatient psychiatric practitioners, such as the outpatient department of psychiatric hospitals, psychotherapists, outpatient neurologists and psychiatrists. Overall, 63 people said they were treated by a general practitioner or internist (33.2%). Other specialties were mentioned less frequently in this context (*n* = 27, 14.3%). Inpatient psychiatry stays were reported 5 times (2.6%). In total, 48 applicants did not specify their contacts to the health care system further (25.3%).

Residency status

In total, 41.0% of the 437 applicants had a temporary residence permit for the time of the asylum request (*n* = 179). A total of 123 (28.2%) persons indicated that they had a temporary suspension of deportation. In total, 90 clients (20.6%) reported that they currently had a residence permit. A total of 32 applicants did not provide any information (7.3%). Among the applicants were also persons who were threatened with deportation because their application was rejected, their residence permit had expired or they were in church sanctuary (*n* = 10, 2.3%). More details are given in Table 4.

Reason for consultation

In addition to the diagnosis of mental disorders and psychotherapy, the PSZ also provides expert opinions and social counselling. The requirement for psychotherapy was the most common motive that led to a consultation with the PSZ (*n* = 345, 79.0%). In 97 cases, a diagnostic order was requested (22.2%). About one-fifth of applicants needed expert opinions (*n* = 94, 21.5%) or social counselling (*n* = 91, 20.8%).

Electronic health insurance card

An electronic health insurance card greatly facilitates asylum seekers’ access to the health system, and allows doctors to charge for the treatment with less administrative effort. Almost half of the clients did not have an electronic health insurance card when registering with the PSZ (*n* = 214, 49%).

Referring Institutions

On 212 registration forms there was information on who had initiated or supported the client’s registration with the PSZ. In total, 64 applications were not submitted by an institution, but by a private person or by the clients themselves (30.2%). A total of 148 registration forms named institutions as the referring party. Non-statutory welfare organizations (*n* = 25, 11.8%), group living quarters (*n* = 17, 8.0%), reception centers for immigrants (*n* = 11, 5.2%) and public authorities (*n* = 10, 4.7%), such as youth welfare offices, prisons or administrative districts were often mentioned. Hospitals also referred their patients to the PSZ (*n* = 11, 5.2%), some of them even coming directly from a psychiatric ward (*n* = 3, 1.4%).

### 3.3. Situation at Assessment Interview

The assessment interviews took place on average 16 weeks after application (min: 0 weeks, max: 175 weeks). Of initially 437 applicants, 188 were seen for the assessment interview while 210 of the applicants could not be reached or stated that they do not need the PSZ’s services any more. In 39 cases, registration forms of assessed clients could not be found.

Torture

Overall, 23 clients explicitly reported experiences of torture without being prompted (12.2%). A total of 20 clients (10.6%) explicitly reported they had not experienced torture. No information concerning torture was documented in 90 files (47.9%) or the subject of torture was not addressed by the client in the interview and this point was documented as ‘not specified’ (*n* = 55, 29.3%). In 77.2% of cases, therefore, we cannot identify any information.

Assessment of urgency

After the interview, the psychologist noted whether this case needed to be treated ‘urgently’ or ‘soon’ or whether it was classified as having an ‘average’ level of urgency.

Overall, 17.6% of the cases to be investigated were classified as ‘urgently’ by the psychologist (*n* = 33). A total of 30.3% of the cases were classified as requiring treatment ‘soon’ (*n* = 57). In total, 52 cases were handled as ‘average’ (27.7%). No data were given in 46 (24.5%) cases.

When stratifying the psychologists’ assessment of urgency according to patients’ gender, women tended to be classified as ‘urgent’ more often than men (24.4% vs. 12.3%).

Stratification for country of origin at the time of the assessment interview showed no relevant differences between subgroups.

On an individual basis, patients’ assessment of their condition’s urgency was later confirmed by psychologists’ classification as ‘urgent’ or ‘soon’ in 24.7% (*n* = 22) and 31.5% (*n* = 28) of the self-professed emergency cases.

The average waiting time from the assessment interview to the start of therapy was 50 weeks. Clients classified as “urgent” had to wait an average of 42 weeks. In contrast, clients who were classified as “soon” in need of treatment had to wait an average of 52 weeks and clients who were classified as “average” urgently had to wait an average of 63 weeks.

Medications

Overall, 99 out of 188 clients mentioned regular medication intake in the interview. Analgesics were most commonly mentioned (*n* = 31, 31.3%), followed by antidepressants (*n* = 28, 28.3%), hypnotics (*n* = 20, 20.2%), neuroleptics (*n* = 10, 10.1%), and proton-pump inhibitors (*n* = 7, 7.1%).

Anticonvulsants (*n* = 3, 3.0%) and benzodiazepines (*n* = 2, 2.0%) were prescribed less frequently. Rarely prescribed medications were summed up as ‘other’ (*n* = 12, 12.1%). Overall, 20 clients were unable to give a specific indication of which medications they were taking.

Disorder-relevant psychopathological symptoms

In total, 85.6% of respondents reported sleep disorders (*n* = 161). These included nightmares as well as disorders of initiating and maintaining sleep. More than half of clients reported pain (*n* = 123, 65.4%), anxiety (*n* = 118, 62.8%), and suicide attempts/suicidal thoughts (*n* = 103, 54.8%). Restlessness, appetite disorders and aggressiveness were each reported by more than one-third of clients. In total, 31 clients reported use of illegal substances (16.5%), 29 hallucinations (15.4%) and 21 self-injuring behavior (11.3%).

Residence status

After a waiting period of an average of 16 weeks, the clients were asked again about their residency status in the assessment interview.

Of the remaining 188 clients, 57 persons reported that they currently have a ‘temporary residence permit’ or a ‘temporary suspension of deportation’ (30.3% each). In total, 13.3% had a ‘residence permit’ (*n* = 25). We do not have any data for 49 clients (26.1%). More details are given in Table 4.

Social support

In total, 74 clients said they fled with their family (39.4%) while 80 people (42.6%) said they were in Germany without companions.

When asked about social support, most of the clients who provided information mentioned family (*n* = 53, 28.2%) or friends and acquaintances (*n* = 50, 26.6%). Volunteers (*n* = 12, 6.4%), religious communities (*n* = 3, 1.6%) or educational institutions (*n* = 5, 2.7%) were mentioned substantially less often. A large proportion of respondents did not provide any information (*n* = 76, 39.4%).

Contacts

In order to provide support to clients with very urgent concerns during the sometimes very long waiting time, they were either referred directly or were provided with contacts of other agencies for the meantime. These contacts were documented on the assessment sheet. Of the 71 contacts that had been recommended, 31 were in counselling centers (41.9%). Doctors and psychologists (*n* = 26, 35.1%), clinics (*n* = 19, 25.7%), and the outpatient department of psychiatric hospitals (*n* = 7, 9.5%), were recommended as well. Reference was also made to special social counselling (*n* = 7, 9.5%) or meeting places (*n* = 5, 6.8%).

### 3.4. Changes during the Time until Assessment

Treatments

Our analysis showed that 14 out of 188 (7.5%) clients had a hospital stay between registration and assessment interview. 76 (40.0%) of the clients had at least one physician contact during the waiting period. On the other hand, this indicates that 60% of clients did not have professional medical support during the 16-week time period between application and interview despite having reported mostly severe complaints at the beginning of the waiting period.

Change in residence status

In 28.2% of the observed people, the residence status did not change during the time until assessment (*n* = 53). 21.3% of the clients experienced a deterioration in their residence status while waiting for an assessment interview (*n* = 40). Six clients (3.2%) improved their residence status, while we did not have sufficient data for 89 clients (47.3%). Table 5 gives an overview.

## 4. Discussion

In 2013, the EU set binding minimal standards for the health care of asylum seekers under Directive 2013/33/EU. All member states of the EU are required to implement the care of vulnerable patient groups into their national law. Previous literature indicates that the requirements of this directive have not been sufficiently met by German law [24] and that there is a gap in the health care system for asylum seekers as compared to the general population [17].

Before this background, the aim of this study was to map the situation of asylum seekers with psychological complaints in Saxony-Anhalt who do not receive treatment within the regular health system but resort to psychosocial services offered by civil society. Our results show that the PSZ’s clientele present mostly with a high level of suffering and a scant support structure.

Building on these findings, in the following we will discuss the most important determinants of asylum seekers’ mental health and access to psychotherapy in the context of the literature and then draw some conclusions for changes to be implemented in the German health care system.

Post-migration stress and resilience

Achieving a residence permit is uncertain and can take a long time. Especially with a temporary suspension of deportation, asylum seekers live in a state of legal limbo, which threatens their well-being. If asylum seekers’ asylum claims are rejected, this represents an additional, existential threat that leads to massive stress [25,26,27]. As our data show, 21% of the PSZ’s clients are subjected to this kind of stressor, while they are still waiting for the assessment interview.

The relationship between mental disorders and post-migration stress is frequently described in the literature [24,26,27]. It is known that post-migration stressors, alone or in combination with previous traumatic stress, often lead to depressive symptoms [28]. Heeren et al. show in their work how residence status contributes to psychopathology [25]. Thus, the deterioration of residence status seen in our data is an additional stressor that threatens to lead to a further aggravation in the mental health of the clients.

Further, concerns about family members who were left behind present a challenge to the mental health of refugees [29,30]. In our data, the high level of post-migration stressors reported by clients waiting for psychotherapy combined with a lack of resilience-supporting resources thus highlight the need for measures to shorten the time until assessment and waiting for therapy, and to support clients while waiting.

Current literature recognizes resilience as the “ability to maintain one’s orientation towards existential purposes despite enduring adversities and stressful events“ [31]. This ability is closely tied to psychological resources. Family and social contacts are particularly important resources for refugees [32,33,34,35]. However, the majority of PSZ clients fled alone. If helpful social support has been reported at all, it was mostly by family or friends and acquaintances. Professional or institutional bodies are rarely mentioned. Sundvall et al. make it clear in their work that combating social exclusion is an essential aspect of promoting mental health: Refugees need support in maintaining or restoring close social networks in order to maintain their well-being and mental health [36]. The reported lack of resources, in turn, represents another source of post-migration stress.

Health insurance card

While the literature describes the handout of Health Insurance Cards immediately after asylum seekers’ arrival as an important step for improving their access to health care [37], less than halve of all clients in our study population had such a card. This low number is due to the fact that the government of Saxony-Anhalt has decided against introducing such a health card for refugees. Bozorgmehr and Razum discuss the effects of restricting access to health care in this manner has on both the clients themselves and the health system. They suggest that restrictions on access to the health system ultimately entail higher costs than unrestricted access [11]. A focus on primary health care for asylum seekers would therefore lower the overall cost of health care [38]. In addition, the symbolic meaning of such a card, which guarantees people constant and unrestricted access to the health system, should be taken into account [39].

Therefore, withholding such a card and the associated discrimination constitutes a further source of stress for refugees [40].

Gottlieb et al. concluded that removing barriers to health care for asylum seekers would not merely benefit asylum seekers themselves. Administrative burdens and ethical tensions would be reduced and financial transparency increased. At the same time, the costs for outpatient health care would be reduced [16].

Handing out a health card to asylum seekers would therefore help them to participate in the German health system on more equal terms and reduce insecurities and the associated stress. The treating physicians, on the other hand, would not have to initiate a separate reimbursement process, which means additional work with unfamiliar processes [14,15].

Navigation in the health system

In our study population, only one-third of clients have a family doctor or internist at the time of registration, and only 31% have had contact with outpatient psychiatric support during the time until assessment. These findings refer to asylum seekers’ problems with navigating the German health care system as it is already described in the literature: Previous studies have shown that asylum seekers face considerable barriers in accessing routine care, mostly due to their insurance status and the bureaucracy it entails [41], but also due to a lack of interpreter services in most sectors of the German health system [42] and discrimination by health personnel [39]. Additionally, the absence of systematic efforts to familiarize asylum seekers with the particularities of the German health system influences their health care utilization and contributes to patterns of utilization which are dysfunctional within the German health system (such as consulting hospital’s outpatient departments for primary health care) [3].

Taking into consideration that refugees tend to receive little specialized treatment and are often not or incorrectly diagnosed [3,18,43], Kaltenbach et al. demand that physicians and mental health specialists work together to ensure the best possible treatment in a timely manner [43]. In the German health system, general practitioners provide a junction where the clients’ health information from different specialties comes together and is holistically assessed. They undertake coordinative and referring tasks and make a significant contribution to the quality of care in the German health system. Lacking this support, asylum seekers often do not receive coordinated and therefore no high-quality care [18,44]. This could explain why in our study population despite the high number of doctor visits, there is still a high level of suffering at the time of the assessment interview, as we can see from the subsection ‘Psychopathologies’ above.

Legal restrictions in conflict with the EU directive

The EU directive clearly demands that the specific situation of vulnerable groups of persons should be taken into account. As expected, our data show that the PSZ’s clientele is mostly composed of seriously mentally ill individuals with high levels of suffering and psychological stress. The urgency of the treatment is rated as high by both clients and professionals. However, instead of being able to rely on important resources such as social support and structural security, low-threshold access to the health system or secure residence status, these are either missing or denied.

Especially the legal framework responsible for this situation deserves special mention here: Instead of implementing inclusive strategies that take into account the vulnerabilities of asylum seekers (as required by the EU), the government provides a health policy framework that asylum seekers and professionals within the health care sector perceive as arduous, bureaucratically inefficient and incapacitating [41] and that excludes them from standard care [13].

What does it take?

In a time of illness, psychosocial stress and a lack of resources to deal with this situation, mentally ill asylum seekers need quick and well-organized support.

Almost 20 years ago, Machleidt and colleagues tried to provide treatment impulses for the psychiatric and psychotherapeutic care of migrants [7,45]. These proposals are still relevant today and we can underline their demands against the background of our results.

Few of the suggestions made at that time have been implemented, while those which have been implemented have proven to be successful, when evaluated: For example, our working group was able to identify good usability and high usefulness of the ‘Health Booklet for Asylum Seekers’ [42]. Furthermore, Machleidt et al. called for improved access to regular health care through low-threshold, culturally sensitive and competent therapy approaches, in which employees with migration background and psychologically trained specialist interpreters are included. In addition, they emphasized the importance of expanding the cooperation between heath care with general practitioners, social services and social associations, such as non-statutory welfare organizations. They also highlighted the targeted intercultural training of service providers and therapists [7,45]. Unfortunately, most of these demands have not been established on a grand scale within the German health care system, even though some hospitals—mostly in metropolitan areas—offer transcultural care, but are often restricted to regularly ensured patients [46].

The PSZs on the other hand, as non-governmental service providers, have specialized in working with asylum seekers for years and implemented these requirements into their working routine. Their work expressly includes interdisciplinary collaboration between psychologists, social workers and other professions and underlines the importance of networking with other actors from the health sector and interpreters [8]. Interculturally trained psychologists and therapists who specialize in the therapy of severely traumatized patients are indispensable for meeting the challenge of providing good patient care. These clinics are often underfunded and lack the resources to meet the huge demand. As a result, asylum seekers have to wait much longer for psychotherapy than patients who are cared for in the regular health system.

Limitations

This study entails a number of limitations that curtail its generalizability. First, it is based on the clients’ charts of one institution. While other PSZs are known to work under similar conditions and face similar challenges [8], health care infrastructure and administrative requirements differ from federal state to federal state in Germany, so that we cannot easily generalize our findings to other parts of Germany. In addition, our sample is rather small, especially the sample of clients for whom we could assess both registration form and assessment interview. Therefore, many subgroup analyses that would have been interesting were not possible or yielded no results.

In addition to those limitations concerning generalizability, the fact that our analysis was built on secondary data should be noted: Complaints, diagnoses and symptoms—to name a few—are derived either from clients’ self-assessment or from anamnesis. The quality of this information is therefore uncertain.

## 5. Conclusions

Our data show that asylum seekers wait substantially longer for psychotherapy than other patients. Two conclusions must therefore be drawn: In the short term, existing civil society services should be strengthened through better government funding. In the long term, the regular health system should increasingly open up to the needs of asylum seekers, which would entail changes on the level of social legislation, medical training and interdisciplinary cooperation.

## Figures and Tables

**Table 1:** Demographic characteristics of the study population.

	Characteristic	*n* = 437	%
Gender	Female Male	171 266	39.1 60.9
Country of origin	Afghanistan	139	31.8
	Syria	56	12.8
	Iran	41	9.4
	Russia	22	5.0
	Somalia	20	4.6
	Iraq	18	4.1
	Chechnya	15	3.4
	Guinea Bissau	14	3.2
	Benin	13	3.0
	Burkina Faso	11	2.5
	Turkey	9	2.1
	Mali	8	1.8
	Kosovo	7	1.6
	Nigeria	7	1.6
	Unknown	8	1.8
	Others	49	11,2
Age group in years	18–19	43	9.8
	20 ≤ 24	98	22.4
	25 ≤ 29	99	22.7
	30 ≤ 34	67	15.3
	35 ≤ 39	52	12
	40 ≤ 44	31	7.1
	45 ≤ 49	20	4.6
	50 ≤ 54	13	3.0
	55 ≤ 60	12	2.8
	>60	2	0.5
	mean = 30.7 years		
Spoken languages	Persian/Farsi/Dari	183	41.9
	English	83	19.0
	German	75	17.2
	Arabic	71	16.3
	French	42	9.6
	Russian	42	9.6
	Pashto	37	8.5
	Kurdish/Sorani	37	8.5
	Somali	21	4.8
	Turkish	18	4.1
	Chechen	15	3.4
	Albanian	7	1.6
	Tigrinya	6	1.4
	Others	58	13.27
Interpreter needed	Yes No	360 77	82.4 17.6

**Table 2:** Absolute and relative frequencies of mentioned complaints.

Complaints	*n* = 437	%
Insomnia	225	51.5
Anxiety	143	32.7
Trauma/PTSD *	84	19.2
Headache	81	18.5
Depression	69	15.8
Suicidal ideation	67	15.3
Nightmares	65	14.9
Depressiveness	55	12.6
Sadness	47	10.8
Stress	45	10.3
Pondering	44	10.1
Pain	42	9.5
Others	36	8.3
Reduced concentration	33	7.6
Unrest	32	7.3
No specific information	32	7.3
Aggressiveness	26	6.0
Social retreat	24	5.5
Memory problems	24	5.5
Reduced appetite	22	5.0
Flashbacks	21	4.8
Self-injury	20	4.6
Exhaustion	19	4.4
Panic	17	3.9
No statement	16	3.7
Dissociation	14	3.2
Worry	13	3.0
Affect lability	11	2.5
Fainting attacks	10	2.3
Stomach discomfort	10	2.3
Consumption of drugs	9	2.1

* Post-traumatic Stress Disorder.

**Table 3:** Emergencies specified on the registration form.

		*n* = 437	%
Emergency specified?	Yes No	207 230	47.4 52.6
		*n* = 207	%
Specified emergencies	Suicidal ideation/Attempted suicide/Self-injury	81	39.1
	Other acute stress situations	28	13.5
	Threatened with/Imminent deportation	21	10.1
	Social indication	20	9.7
	chronic condition	16	7.7
	Exacerbation	8	3.9
	Severe disease/poor acute condition	6	2.9
	Follow-up therapy	5	2.4
	Pain	3	1.5
	Court order for treatment	1	0.5
	No statement	18	8.7

**Table 4:** Residence status at the time of registration and the initial assessment interview.

	Registration		Assessment Interview	
	*n* = 437	%	*n* = 188	%
Temporary residence permit *(Gestattung)*	179	41.0	57	30.3
Temporary suspension of deportation *(Duldung)*	123	28.2	57	30.3
Residence permit *(Aufenthaltserlaubnis)*	90	20.6	25	13.3
No data	32	7.3	49	26.1
Other	13	3.0		
Rejected application	3	0.7		
Without residential status	3	0.7		
Permit expired	2	0.5		
Dublin returnees	2	0.5		
Threatened with deportation/ Imminent deportation	1	0.2		
EU migrant	1	0.2		
Church sanctuary	1	0.2		

**Table 5:** Change in residence status in the time between registration and assessment interview.

	*n* = 188	%
Improvement	6	3.2
Constancy	53	28.2
Deterioration	40	21.3
No Data	89	47.3

## Data Availability

Data for this research were used under license and are therefore not publicly available. Data are, however, available from the authors upon reasonable request and with permission of the PSZ Halle, Germany.

## Supplementary Materials

The following are available online at https://www.mdpi.com/article/10.3390/ijerph182211850/s1. Table S1: Diagnoses encoded according to the ICD-10.
