# Supplementary material for: Sick and Tired—Sociodemographic and Psychosocial Characteristics of Asylum Seekers Awaiting an Appointment for Psychotherapy
Source: Int J Environ Res Public Health. 2021 Nov 12;18(22):11850. doi: 10.3390/ijerph182211850 (PMC8619663; doi:10.3390/ijerph182211850)
Supplement: Supplementary file 1 [file ijerph-18-11850-s001.zip › ijerph-1416518-supplementary.pdf]

Table S1. Diagnoses encoded according to the ICD-10.

Diagnoses	Stating *n* = 437	%	Total *n* = 141	%
**F10.1**	**3**	2.1	3	0.7
**F10.2**	1	0.7	1	0.2
F12.1	2	1.4	2	0.5
F13.2	2	1.4	2	0.5
F15.1	1	0.7	1	0.2
F20.0	2	1.4	2	0.5
F20.9	1	0.7	1	0.2
F23.8	1	0.7	1	0.2
F29	1	0.7	1	0.2
F31.1	1	0.7	1	0.2
F32.1	12	8.5	12	2.8
F32.2	10	7.1	10	2.3
F32.2 V.a.	1	0.7	1	0.2
F32.9	23	16.3	23	5.3
F32.9 V.a.	3	2.1	3	0.7
F33.1	2	1.4	2	0.5
F33.2	3	2.1	3	0.7
F33.3	2	1.4	2	0.5
F33.9	2	1.4	2	0.5
F34	1	0.7	1	0.2
F40.9 V.a.	1	0.7	1	0.2
F40.9	1	0.7	1	0.2
F41	7	5.0	7	1.6
F41 V.a.	1	0.7	1	0.2
F41.1	2	1.4	2	0.5
F41.2	3	2.1	3	0.7
F41.9	1	0.7	1	0.2
F42.0	9	6.4	9	2.1
F43.0	8	5.7	8	1.8
F43.1	63	44.7	63	14.4
F43.1 V.a.	3	2.1	3	0.7
F43.2	15	10.6	15	3.4
F43.9	2	1.4	2	0.5
F44.9	5	3.6	5	1.1
F45.1	1	0.7	1	0.2
F45.9	3	2.1	3	0.7
F45.9 V.a.	2	1.4	2	0.5
F51.9	6	4.3	6	1.4
F60.31	1	0.7	1	0.2
F60.4	1	0.7	1	0.2
F81	1	0.7	1	0.2
F84.1	1	0.7	1	0.2
F91.9	1	0.7	1	0.2
